# A Poor Prognostic Case of Mucoepidermoid Carcinoma of the Thyroid: A Case Report

**DOI:** 10.1155/2012/862545

**Published:** 2012-08-30

**Authors:** Koji Shindo, Shinichi Aishima, Masayuki Okido, Akira Ohshima

**Affiliations:** ^1^Department of Surgery and Oncology, Graduate School of Medical Sciences, Kyushu University, 3-1-1 Maidashi, Higashi-ku, Fukuoka 812-8582, Japan; ^2^Department of Anatomic Pathology, Graduate School of Medical Sciences, Kyushu University, 3-1-1 Maidashi, Higashi-ku, Fukuoka 812-8582, Japan; ^3^Department of Surgery, Hamanomachi Hospital, 3-5-27 Maizuru, Chuo-ku, Fukuoka 810-8539, Japan; ^4^Department of Surgery, Ito Clinic, 265-1 Hatae, Itoshimashi, Fukuoka 819-1104, Japan

## Abstract

Mucoepidermoid carcinoma (MEC) of the thyroid is very rare and low-grade indolent neoplasm. In past reports of the thyroid MEC, only seven cases were described as poor prognosis. A 91-year-old woman presented with a rapidly growing mass of the left upper neck. She was followed thyroid papillary carcinoma (PC) without operation for two years. Fine needle aspiration cytology (FNAC) showed undifferentiated cells. Total thyroidectomy and bilateral neck dissection were performed. In pathological findings, the tumor had two areas of MEC and PC. The boundary of them was mixed. She died of multiple lung metastases only after four months from the operation. We report a rare case of thyroid MEC which had an aggressive behavior and poor prognosis. This case is a precious in that thyroid MEC occurred during observation of PC and suggests a possibility of the transformation from PC to MEC.

## 1. Introduction

Mucoepidermoid carcinoma (MEC) is a common neoplasm of the salivary gland but can also occur in other organs such as esophagus, breast, lung, pancreas, and thyroid gland [1–5]. MEC is a very rare variant of thyroid cancer, and about 40 cases have been reported to date. Many authors assumed it as a low-grade neoplasm, in which prognosis was not so dismal [6–8]. According to the past reports, only seven cases of thyroid MEC with poor prognosis were described [9–14]. We herein report a case of thyroid MEC that occurred during followup of papillary carcinoma, which behaved aggressively, and died four months after surgery.

## 2. Case Report

A 91-year-old woman presented with a chief complaint of rapidly growing mass of the left upper neck with pain. Two years before she had a mass about 3 cm diameter at her left neck, and hypothyroidism was pointed out. Treatment for hypothyroidism with the administration of levothyroxine sodium hydrate was started. The mass was in the left lobe of the thyroid gland and diagnosed as a papillary carcinoma by fine needle aspiration cytology (FNAC). A regional nodal metastasis had been also pointed out. Her family had declined operation for her advanced age. However, the mass was rapidly growing with pain during a last month.

On physical examination, a large hard mass measuring 5 cm was located at left upper neck, and two skin implantations were also seen at the site of FNAC performed two weeks before. Laryngoscopy showed a left vocal cord paresis.

Thyroid function tests were performed: serum thyroid stimulation hormone (TSH) 0.064 *μ*IU/mL (0.27–4.20 *μ*IU/mL) was very low, because of feedback from medication for hypothyroidism, free serum triiodothyroxine (FT3) 1.97 pg/mL (2.60–5.10 pg/mL), free serum thyroxine (FT4) 1.22 ng/dL (1.00–1.80 ng/dL), and serum thyroglobulin 115 ng/mL (less than 30 ng/mL). Carcinoembryonic antigen (CEA) 1.0 ng/mL was not elevated. And other routine laboratory data showed no abnormal findings.

**Figure 1:**
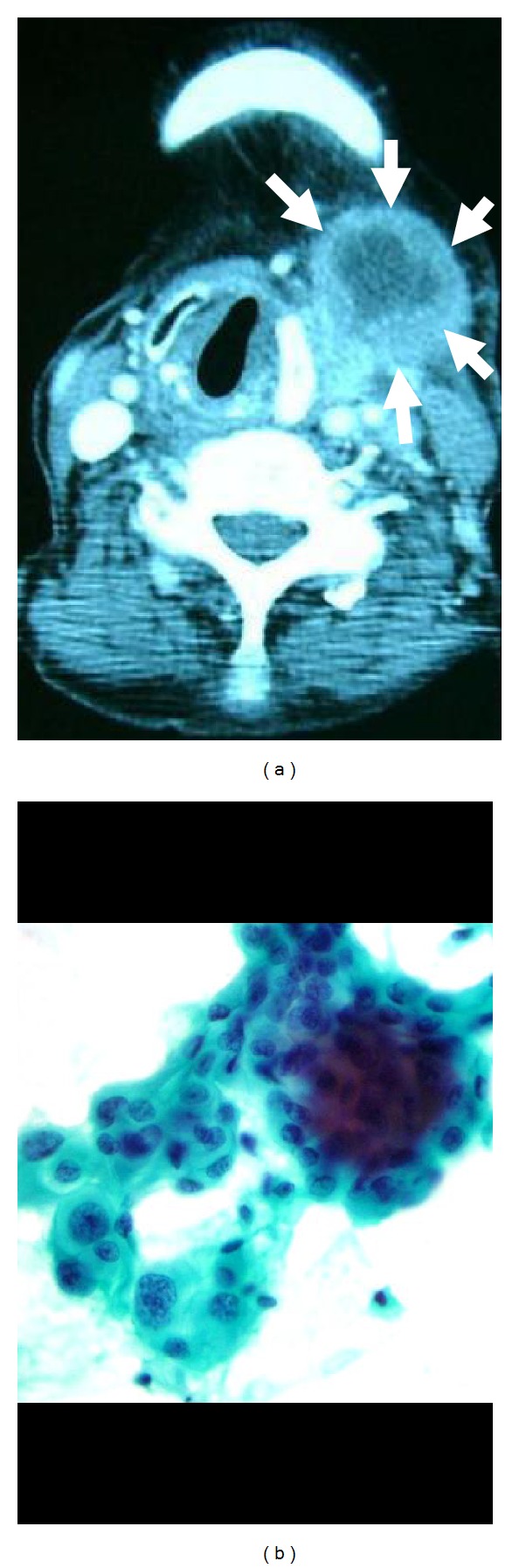
(a) Enhanced computed tomography (CT) revealed a large tumor with internal hypodensity lesion (arrow). (b) Fine needle aspiration cytology (FNAC) of the left large tumor revealed a proliferation of pleomorphic cells with abundant cytoplasm showing clear-cut and atypical features (Papanicolaou staining).

Ultrasonography showed a large tumor with hypoechoic area at left upper neck and another tumor, 2 cm in size, in right lobe accompanied by some enlarged lymph nodes along both jugular veins. Enhanced computed tomography (CT) suggested that the left neck tumor invaded to the left jugular vein and trachea, which displaced to right (Figure 1(a)). Distant metastasis was not recognized.

FNAC of the left tumor revealed a proliferation of pleomorphic cells with abundant cytoplasm showing clear-cut and atypical features, suggesting undifferentiated malignant cells (Figure 1(b)). Another tumor in the right lobe was showed papillary carcinoma (PC) by FNAC. The patient underwent the operation to avoid suffocation and other local complication.

**Figure 2:**
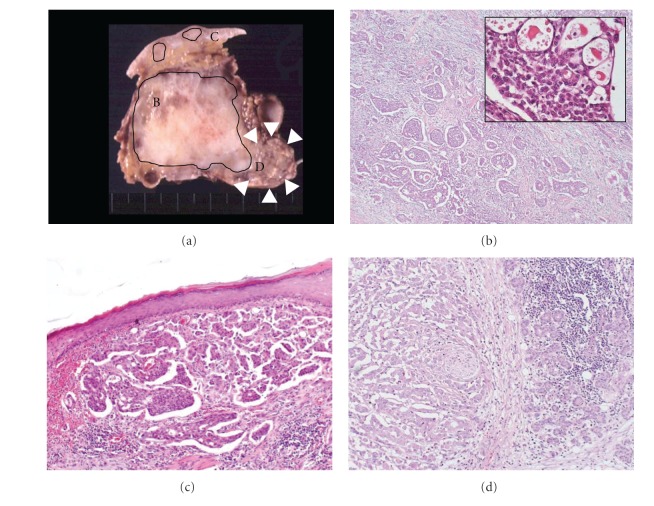
(a) Cut surface at maximal diameter. Upper tumor was close to the submandibular gland (arrowhead). The area of curved line indicates MEC. (b–d) Hematoxylin and eosin (HE) staining (original magnifications: (b) ×40 (inset ×200), (c), (d) ×100). (b) Upper tumor showed cellular islands, in which tumor cells were squamoid while other showed mucin secretion. (c) The site of skin implantation was caused by MEC. (d) MEC and submandibular gland were separated by fibrous tissue.

**Figure 3:**
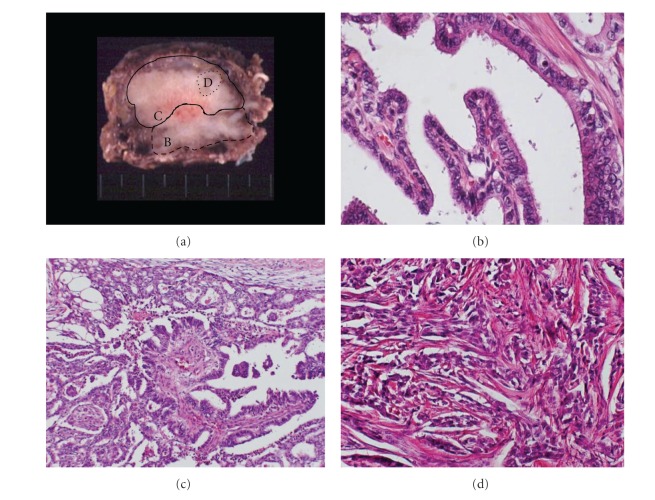
(a) Cut surface at 1 cm lower from maximal diameter. Each lined area indicates MEC (curved line), PC (dashed line), and poorly differentiated component (dotted line). (b–d) Hematoxylin and eosin (HE) staining (original magnifications: (b), (d) ×200, (c) ×100). (b) Lower tumor showed typical papillary carcinoma. (c) The boundary of MEC and PC was mixed. (d) Poorly differentiated component was seen in a small area.

In surgical findings, the left tumor invaded the surrounding muscles, submandibular gland, thyroid cartilage, cricoid cartilage, and trachea. And enlarged lymph nodes were attached to the left jugular vein, thoracic duct, and bilateral recurrent nerves. We performed total thyroidectomy, bilateral neck dissection, and resections of left submandibular gland and left jugular vein. Shaving was performed at the invaded surface of trachea and bilateral recurrent nerves and preserved them. Tracheostomy was performed to make sure the breath control. The resected specimen had internal necrosis and infiltrated into surrounding tissues, including left submandibular gland, isthmus of the thyroid gland, and dermal tissue along the wounds of cytology (Figures 2(a) and 3(a)).

In pathological findings, the left tumor had two components divided to upper site and lower site. Upper site showed MEC (Figure 2(b)), and lower site showed PC (Figure 3(b)). The tumor cells of MEC were arranged in solid and cribriform pattern, with elongated lumina containing colloid-like material, and had no any association of sclerosis or eosinophils (Figure 2(b)). Immunohistochemically, the carcinoma cells were positive for thyroglobulin, and cytokeratin 903 in squamoid cell component, and mucin was confirmed by Alcian blue. In addition, immunohistochemical reaction for TTF-1 was weakly positive at the PC area, but inconspicuous at the MEC area, neither mucinous nor squamous component in this case. Skin implantation was caused by MEC (Figure 2(c)). MEC and submandibular gland were separated by fibrous tissue (Figure 2(d)). The boundary of MEC and PC was mixed (Figure 3(c)). The lower site of MEC contained a little (7 mm in size) poorly differentiated component (Figure 3(d)). The tumor of the right lobe showed PC only. PC invaded muscles, cartilage, trachea, and cervical vein. Lymph nodes were metastasized by both carcinomas.

She tried external radiation therapy but discontinued because of odynophagia and radiation dermatitis, with a total dose of 27 Gy. After two months from the operation, CT revealed multiple lung metastases and thoracic lymph node metastases. She died after four months from the operation.

## 3. Discussion

MEC is one of the common neoplasms occurring in salivary gland. According to the literature about salivary tumor, MEC comprises about 10% of all salivary gland neoplasms and about 35% of malignant ones [15]. However, MEC can also occur in other organs such as esophagus, breast, lung, pancreas, and thyroid gland [1–5]. It rarely occurs in thyroid gland, and about 40 cases of thyroid MEC have been described since first report in 1977 by Rhatigan et al. [5].

The histogenesis of thyroid MEC has been widely debated in the literature. Two major conceivable origins are thyroid follicular epithelial cell [6, 14, 16, 17] and the cell of solid cell nests (SCN) [11, 18–20], which is regarded as the vestiges of the ultimobranchial body. Evidence supporting SCN origin is histologic similarities between MEC and SCN [9, 19], and existence of ductal structures lined by ciliated columnar epithelial cells in both [18, 20]. In addition, SCN can have multipotential cells derived from the ultimobranchial apparatus and can contribute to generate various thyroid cancers including papillary carcinoma and MEC [10, 18, 20]. Also, this hypothesis is supported by the existence of some cases that had combined MEC and papillary carcinoma of the thyroid [10, 11].

On the other hand, the fact of the existence of combined MEC and PC is also described as the evidence supporting follicular epithelial cells origin [21–23]. Some authors regarded the coexistence of them as an evidence for same origin, and a papillary carcinoma occurs from follicular epithelium as we know. They also considered that MEC might arise from preexisting papillary carcinoma, triggered by squamous and mucinous metaplasia [6, 7]. Moreover, thyroid specific mRNAs such as *TTF-1* and *PAX-8* were detected simultaneously in metastatic axillary lymph node of MEC [14]. These two mRNAs are present together only in thyroid follicular cells.

MEC of the thyroid gland with anaplastic lesion has been reported in four cases to date, and all of them had poor prognosis [9, 11–13]. On the other hand, papillary carcinoma combined with MEC is relatively common [11, 16, 24]. In our case, the tumor revealed PC by FNAC that was stable for two years and rapidly growing with MEC. MEC and PC were mixed histopathologically. These findings show a possibility of transformation from PC to MEC. In addition, the mass was mostly occupied by MEC and PC, and poorly differentiated component was only in a small area at the lower site of MEC. These findings indicate that the aggressive behavior of this case did not depend on that poorly differentiated component.

The five-year survival rate of MEC occurred in salivary gland that is nearly 90%, and it is considered low-grade malignancy [25]. The prognosis of thyroid MEC is almost good as well as salivary gland, but seven cases were reported as poor prognostic disease to date (Table 1). Two cases of thyroid MEC had not underwent operation because of their aggressiveness [12, 14]. All of seven cases died within only 13 months despite of multidisciplinary treatment such as radiation, chemotherapy, and ^131^I. In pathological findings, all of them had papillary carcinoma with/without variant formation [9–11, 13, 14] or anaplastic lesion [11–13] in MEC. In our case, lung and thoracic lymph node metastasis appeared after two months from the operation and caused death after four months. It is possible that these aggressive cases were caused by transformation from PC to MEC.

Among all the reported cases except these seven cases, some other cases were also described as aggressive behavior with invasion, metastases, and recurrence [20, 21, 24]. Most of these aggressive cases had lymph nodes metastasis or invasive MEC at the time of operation and had undergone thyroidectomy and lymph nodes dissection [22]. Those cases had been added other procedures such as radiation, chemotherapy, and ^131^I and survived longer. Instead, all of them were not effective in poor prognostic cases. Still it is difficult to distinguish good prognostic cases from poor one. Chemoradiation should be considered for the aggressive cases.

## 4. Conclusion

We report an aggressive and poor prognostic case of MEC of the thyroid. MEC was assumed as a low-grade neoplasm, in which prognosis was not so dismal, but sometimes had aggressive behavior. We should take a careful followup. In addition, this is a precious case in that thyroid MEC occurred during followup of PC. This case suggests a possibility of the transformation from PC to MEC.

## Figures and Tables

**Table 1:** Patient information of poor prognostic thyroid MEC (*n* = 7).

Age/sex (citation)	Survive	Accompanied tumor	Recurrence	Treatments
54/F [9]	13 mo	PC anaplastic ca.	Neck lymph nodes	Operation XRT + Chem
66/F [10]	11 mo	PC	Neck	Operation XRT + I + Chem
62/F [11]	10 mo	PC anaplastic ca.	Neck	Operation Chem + I + XRT
57/M [12]	4 wk	Anaplastic lesion		Chem
64/M [13]	3 mo	PC tall cell variant	NA	Operation
83/F [13]	5 mo	Anaplastic ca.	NA	Operation
52/M [14]	2 mo	Variant type of PC		XRT + Chem

mo: months, wk: weeks, NA: not applicable, XRT: X-ray therapy, Chem: chemotherapy, I: ^131^iodine.
